# A Perfect Solution of Salicylic Acid

**Published:** 1880-08

**Authors:** 


A Perfect Solution of Salicylic Acid—

R.—Salicylic acid..................℥ i–℈ viii
Citrate of potash..............℥ ii
Glycerin.......................℥ iii
Simple elixir, q. s. to make....O i

The citrate is to be dissolved in glycerin by the aid of a little heat, after which the acid is to be stirred in and a gentle heat maintained until it is completely dissolved. On cooling, simple elixir is to be added to bring it up to the required measurement. The solution is then to be strained, and when prepared with a colorless elixir is of the color of a very pale cherry. It contains five grains of salicylic acid to the flui-drachm, and is miscible in all proportions with water without the separation of any acid.

The above appears in the Louisville News, the editor of which says it is "the best solution of salicylic acid he has ever used."—*Ohio Medical Recorder.*

